# Crosstalk Between Pyroptosis and Apoptosis in Hepatitis C Virus-induced Cell Death

**DOI:** 10.3389/fimmu.2022.788138

**Published:** 2022-02-14

**Authors:** Hannah L. Wallace, Lingyan Wang, Cassandra L. Gardner, Christopher P. Corkum, Michael D. Grant, Kensuke Hirasawa, Rodney S. Russell

**Affiliations:** ^1^ Immunology and Infectious Diseases, Division of Biomedical Sciences, Faculty of Medicine, Memorial University, St. John’s, NL, Canada; ^2^ Confocal Imaging/Flow Cytometry Unit, Medical Laboratories, Faculty of Medicine, Memorial University, St. John’s, NL, Canada

**Keywords:** pyroptosis, inflammasome, NLRP3, hepatitis C virus, HCV, apoptosis, programmed cell death, pathogenesis

## Abstract

Extensive inflammation in the liver is known to contribute to the pathogenesis of hepatitis C virus (HCV) infection. Apoptosis has, for a long time, been known to act as a mechanism of hepatocyte death, but our previous research also identified inflammasome-mediated pyroptosis in infected and uninfected bystander cells as an additional mechanism of HCV-induced cytopathicity. The purpose of this study was to investigate the mechanism of HCV-induced cell death and to determine the timing and relative contributions of apoptosis and pyroptosis during HCV infection. In a model employing a cell culture-adapted strain of JFH-1 HCV and Huh-7.5 hepatocyte-like cells, we found that pyroptosis occurred earlier than did apoptosis during infection. CRISPR knockout of NLRP3 resulted in decreased caspase-1 activation, but not complete elimination, indicating multiple sensors are likely involved in HCV-induced pyroptosis. Knockout of gasdermin-D resulted in increased activation of apoptosis-related caspase-3, suggesting potential crosstalk between the two cell death pathways. An unexpected decrease in activated caspase-1 levels was observed when caspase-3 was knocked out, implying that caspase-3 may have a role in the initiation of pyroptosis, at least in the context of HCV infection. Lower viral titres in culture fluids and increased ratios of intracellular to extracellular levels of infectious virus were observed in knockout versus wild-type Huh-7.5 cells, suggesting that HCV may induce programmed cell death in order to enhance virus release from infected cells. These results contribute to the understanding of HCV pathogenesis and add to the increasing volume of literature suggesting various programmed cell death pathways are not mutually exclusive.

## Introduction

Multiple forms of programmed cell death (PCD) have been demonstrated in the context of virus infection and it is generally believed that viruses utilize these mechanisms to induce disease (1–4). However, some reports detail how PCD is employed as a host response to contain the virus [reviewed in reference (5)]. Both apoptosis and pyroptosis have been implicated in both contexts.

Apoptosis is a non-inflammatory form of cell death that is mediated by executioner caspase-3 and can be initiated *via* one of two pathways. The intrinsic pathway begins intracellularly in response to changes in the intracellular environment, including, but not limited to, mitochondrial or DNA damage, endoplasmic reticulum stress, or reactive oxygen species, resulting in formation of the apoptosome. In contrast, the extrinsic pathway depends on initiation by ligand-binding of a death receptor on the cell surface, leading to cleavage of caspase-8. Both pathways ultimately result in activation of executioner caspase-3 which leads to cell shrinkage, condensation of chromatin, nuclear fragmentation, and formation of apoptotic bodies that are cleared by circulating macrophages [both pathways extensively reviewed in reference (6)]. Apoptosis has been described as immunologically silent while still having a role in pathogenesis induced by some viruses (7, 8).

Pyroptosis is mediated by an inflammasome, a protein-complex consisting of a sensor, such as NLRP3 [nucleotide oligomerisation domain, leucine-rich repeat, pyrin-domain containing protein 3; (9)] or AIM2 (absent in melanoma 2), an adaptor (ASC; apoptosis-associated speck-like protein containing a CARD [caspase recruitment domain]), and caspase-1 as the effector enzyme (10). Once assembled into an inflammasome, activated caspase-1 cleaves gasdermin-D (GSDM-D) into its mature, pore-inducing form, which ultimately facilitates cell swelling and subsequent cell lysis (11). Pyroptosis, in contrast to apoptosis, is considered to be pro-inflammatory and immunogenic (6). Classically, pyroptosis was thought to function only as an innate immunity mechanism [reviewed in reference (12)], although recent findings suggest pyroptosis may also play a role in viral pathogenesis (3, 13, 14).

Despite availability of highly effective curative drug therapies for treating HCV infection, as many as 71 million people worldwide remain chronically infected, with many of these people unaware of their infected status (15). Some infected individuals who undergo direct-acting antiviral (DAA) therapy still develop worsening liver disease, including hepatocellular carcinoma, despite prior elimination of the virus (16, 17). Plasma levels of many inflammatory cytokines decrease following DAA treatment, with notable exceptions being pyroptosis-associated interleukin-18 (IL-18) and interleukin-1*β* [IL-1*β*; (18)]. It is important to understand how this virus induces liver disease as the burden of HCV on healthcare is predicted to increase in the coming decade (19).

It is well established that non-inflammatory, caspase-3-mediated apoptosis occurs in the context of HCV infection both *in vitro* (20, 21) and *in vivo* (22, 23), and that this form of cell death contributes to liver pathology associated with chronic HCV infection (23). Our group has previously demonstrated that hepatocyte-like Huh-7.5 cells undergo both apoptosis and pyroptosis when infected with cell culture-adapted HCV (HCVcc). We demonstrated involvement of the NLRP3 inflammasome as indicated by a decrease in pyroptotic cell death induced by HCV when infection occurred in the presence of an NLRP3 inhibitor [MCC950; (24)]. To follow up on these findings, the current study aimed to identify the relative timing of these forms of programmed cell death during HCV infection (determining if pyroptosis and apoptosis occur sequentially or concurrently), to confirm the involvement of pyroptosis-associated proteins NLRP3 and GSDM-D and apoptosis-associated caspase-3, and to determine whether programmed cell death has a role in viral spread during HCV infection *in vitro*.

## Materials and Methods

### Cell Culture

Huh-7.5 cells (gift from Apath, LLC) were maintained at 37°C with 5% CO_2_ in complete medium (CM) containing Dulbecco’s Modified Eagle Medium (DMEM; with high glucose [4.5 g/L] and pyruvate; ThermoFisher Scientific, 11995073), supplemented with 10% fetal bovine serum (FBS; heat-inactivated, ThermoFisher Scientific, 10438034) and 1% penicillin/streptomycin (Millipore Sigma, P4333-100ML).

### Virus Stocks

A cell culture-adapted strain of HCV, known as JFH1_T_ (25, 26), was used for this study. To generate virus stocks, 1x10^6^ Huh-7.5 cells were seeded in 10-cm culture dishes. Approximately 24 h later, cells were inoculated at a multiplicity of infection (MOI) of 1 and incubated for 3 h, after which inoculum was replaced with fresh CM. Culture fluids were harvested three days post-infection (p.i.) and virus titre was determined using a limiting dilution focus-forming assay described below.

### CRISPR-Cas9-Mediated Genome Editing

Single guide RNA (sgRNA) sequences were designed to knock out *NLRP3* (NLRP3_F 5’-GCGAAGCAGCACTCATGCGAG-3’, NLRP3_R 5’-CTCGCATGAGTGCTGCTTCGC-3’), *GSDM-D* (GSDM-D_F 5’-GCAGCGAGTACACATTCATTG-3’, GSDM-D_R 5’-CAATGAATGTGTGTACTCGCTGC-3’), or *caspase-3* (CASP-3_F_5’-GTGAGTTTTCAGTGTTCTCCA-3’, CASP-3_R_5’- TGGAGAACACTGAAAACTCAC-3’). Oligonucleotide pairs were annealed and ligated into the BbsI site of a psPCas9-2A-GFP backbone using a rapid DNA ligation kit (Roche, 11635379001). Plasmids were transformed into *Escherichia coli* (DH5α competent *E. coli*; ThermoFisher Scientific, 18258012) and cultures were incubated overnight at 37°C in LB medium, supplemented with 100 µg/mL ampicillin. Plasmids were isolated and transfected into Huh-7.5 cells using Lipofectamine 3000 (Thermo Fisher, L3000075). Transfected cells were selected by fluorescence-activated cell sorting based on GFP expression. Cells were seeded by limiting dilution and knockout clones were expanded.

### Immunofluorescence Microscopy

Cells were seeded at a density of 1x10^5^ cells per well in 2-well chamber slides (Fisher Scientific, 12-565-16). The following day, cells were infected with HCV at an MOI = 1 or left uninfected. Cells were left for one, two, three, or four days following infection for time course experiments or left for three or four days for non-time course experiments. Caspase-1 was visualised using a FAM-FLICA caspase-1 inhibitor kit (ImmunoChemistry Technologies, product number 98), referred to as a caspase-1 probe (27–31). On the designated day, CM was removed from cells and replaced with 30X FAM-FLICA caspase-1 probe in CM. Probe was incubated with cells for 45 min at 37°C. Following incubation, an additional 400 µL of CM was added and cells were incubated for one additional hour. Culture fluids were removed, replaced with a fresh 200 µL of CM, and the cells incubated for 5 min. Cells were then washed in their respective wells with 300 µL of apoptosis wash buffer (ImmunoChemistry Technologies, product number 98) for 20 min at room temperature in the dark. Following washing, cells were fixed and permeabilised by treating each well with 200 µL of 100% acetone for 45 sec at room temperature. After fixation, slides were washed for 1 min in phosphate-buffered saline (PBS; pH = 7.4). The same fixation protocol was used for all staining, independent of caspase-1 probe use. If the probe was not used, cells were fixed prior to staining. HCV core protein and cleaved caspase-3 were detected using anti-HCV core (1:200 dilution in 5% bovine serum albumin [BSA] in PBS; Anogen, product MO-I40015B) and anti-caspase-3 antibodies (1:200 dilution in 5% BSA in PBS; BD Biosciences, 559565), respectively. When the anti-HCV core antibody was used alone, slides were incubated for 20 min at room temperature. If the anti-caspase-3 antibody was used either alone or in conjunction with the anti-HCV core antibody, slides were incubated overnight at 4°C. Following incubation with primary antibodies, slides were rinsed in PBS for 5 min prior to a 20-min incubation with secondary antibodies. For regular flourescence microscopy, when either the anti-HCV core or the anti-caspase-3 antibodies were used with the caspase-1 probe, goat anti-mouse or goat anti-rabbit Alexa Fluor^®^ 594 (Invitrogen, A-11020 or A-11037), respectively, was used as the secondary antibody. If the anti-HCV core antibody was used without the caspase-1 probe, a goat anti-mouse Alexa Fluor^®^ 488 (Invitrogen, A-11029) secondary antibody was used. For confocal microscopy, when both the anti-caspase-3 and anti-HCV core antibodies were used together, goat anti-mouse Alexa Fluor^®^ 647 (Invitrogen, A32728) was used for HCV core and goat anti-rabbit Alexa Fluor^®^ 594 (Invitrogen, A-11037) was used for caspase-3. For cells stained with specific antibodies for cleaved GSDM-D (Asp275, Cell Signaling Technology, 36425S, used at 1:100 dilution) and HCV core (same as above), cells were incubated with the primary antibodies in 5% BSA in PBS overnight at 4°C. The next day, cells were stained with goat anti-rabbit Alexa Fluor^®^ 488 (Thermofisher, A11008) and a goat anti-mouse Alexa Fluor^®^ 594 (Invitrogen, A-11020) antibodies to detect cleaved GSDM-D and HCV core protein, respectively. For cells stained for cleaved poly (ADP-ribose) polymerase (PARP; Asp214, Cell Signaling Technology, D64E10, used at 1:100 dilution) and caspase-3 (same as above), cells were also incubated overnight under the same conditions. The next day, cells were stained using goat anti-rabbit Alexa Fluor^®^ 488 (Thermofisher, A11008) and goat anti-mouse Alexa Fluor^®^ 594 (Invitrogen, A-11020) secondary antibodies to visualize cleaved PARP and caspase-3, respectively. All secondary antibodies were used at 1:100 dilution in PBS. An additional 5-min wash in PBS was performed following secondary antibody staining for all conditions. Slides were mounted using Vectashield Hard Set mounting medium containing DAPI (Vecta Shield Mounting Media, Vector Laboratories, MJS BioLynx Inc., VECTH1400 or Vecta Shield Vibrance Mounting Media, VECTH180010). Slides were viewed using either a Zeiss Axio Imager.M2 immunofluorescence microscope or an Olympus Fluoview FV1000 laser scanning microscope or a Zeiss LSM 900 with Airyscan microscope.

### Western Blotting

**Figure 1:**
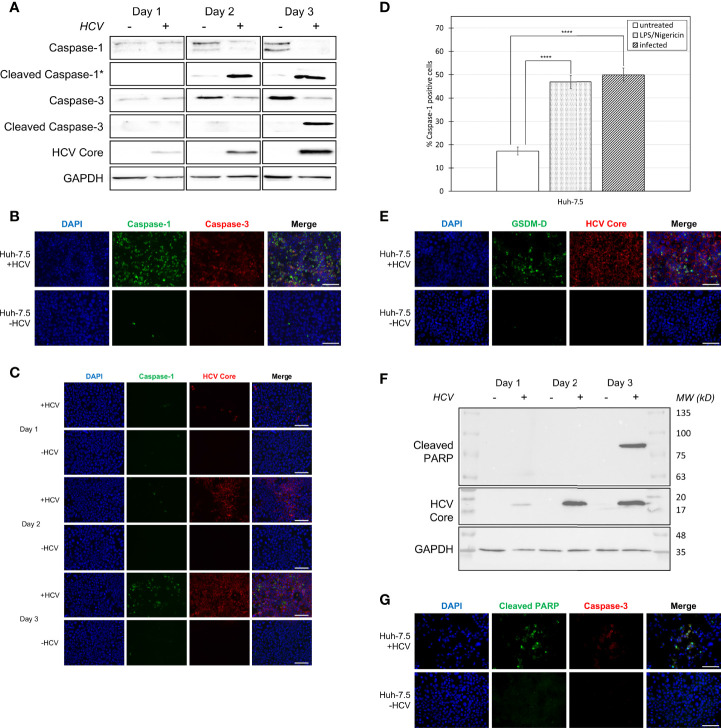
Activation of pyroptosis- and apoptosis-associated proteins during HCV infection. Huh-7.5 cells were infected with HCV at MOI = 1 or left uninfected. **(A)** At 1, 2, and 3 days p.i., cells and culture fluids were harvested for Western Blot analysis. Membranes were probed for pro-caspase-1, cleaved-caspase-1, pro-caspase-3, cleaved-caspase-3, HCV core protein, and GAPDH. * indicates samples from cell culture fluids rather than cell lysates. **(B)** At 3 days p.i., cells were stained for cleaved caspase-1 (green) using a specific probe, then fixed using acetone. Cells were subsequently stained using antibodies specific for cleaved caspase-3 (red). Scale bar, 100 μm **(C)** At 1, 2, and 3 days p.i., cells were stained for cleaved caspase-1 using the same specific probe (green) then fixed using acetone. Cells were subsequently stained using an antibody specific for HCV core protein (red). **(B, C)** Nuclei were stained with DAPI (blue) and analysis was performed using fluorescent microscopy. Scale bar, 100 μm **(D)** At day 4 p.i., cells were stained for cleaved caspase-1 using a specific probe and fixed using fixative from the caspase-1 probe kit. Cells were run on a CytoFLEX flow cytometer and data were analyzed using Kaluza analysis software. Data are presented as the percent of total cells that were positive for caspase-1. ****p < 0.0001. **(E)** At 3 days p.i., cells were fixed using acetone and stained for cleaved GSDM-D (green) and HCV core protein (red) using specific antibodies. Scale bar, 100 μm **(F)** At 1, 2, and 3 days p.i., cells were harvested for Western Blot analysis. Membranes were probed for cleaved PARP, HCV core protein, and GAPDH. **(G)** At 3 days p.i., cells were fixed using acetone and stained for cleaved PARP (green) and cleaved caspase-3 (red) using specific antibodies. Scale bar, 100 μm **(A–G)** Data are representative of three independent experiments.

Cells were seeded at a density of 1x10^6^ in 10-cm culture dishes. On the following day, cells were infected at an MOI = 1 or 0.1, or left uninfected. Cells were then incubated for the desired period of time (one, two, three, or four days p.i.). On the designated day, cells were lysed with RIPA buffer containing protease inhibitors and mixed with loading buffer. Culture fluids were collected and concentrated by centrifugation using Millipore filters (Millipore, Amicon Ultra-4 centrifugal filter units, Ultracel–10K, UFC801096). Prepared samples were heated at 100°C in a heating block for 5 min. Proteins were separated by SDS-PAGE and transferred to nitrocellulose membranes (Amersham/Cytiva, 10600065). Membranes were blocked with 5% skimmed milk powder in TBST (1X TBS with 0.05% Tween-20) for 1 h at room temperature with agitation. Membranes were then incubated with antibodies recognising pro-caspase-3 (Santa Cruz, sc7272), cleaved caspase-3 (Cell Signaling Technology, 9664), pro-caspase-1 (Santa Cruz, sc56036), cleaved caspase-1 (AdipoGen, AG-20B-0048), HCV core protein (Anogen, MO-I40015B or C7-50, Thermofisher, MA1-080), GSDM-D (Santa Cruz, sc81868), cleaved PARP (Asp214, Cell Signaling Technology, D64E10), or GAPDH (Abcam, ab8245) overnight at 4°C at a 1:1000 dilution in TBST. On the following day, membranes were washed in TBST. HRP-conjugated anti-mouse (Santa Cruz, sc516102) or anti-rabbit (Santa Cruz, sc2357) secondary antibodies were incubated with the membranes at room temperature at a 1:5000 dilution in TBST for 1 h. Signal was detected using ECL Western blotting substrates (Clarity Western ECL substrate, Bio-Rad, 170-5060; Amersham ECL select substrate GE Healthcare, RPN2235). All Western blot figures, with the exception of **Figure 1F**, were prepared from images obtained using ImageQuant LAS 4000 (GE Healthcare). The images in **Figure 1F** were obtained using Bio-Rad ChemiDoc Imaging System and processed using Image Lab (version 6.1.0, Bio-Rad Laboratories, Inc.).

### Flow Cytometry

Cells were seeded at a density of 1.5x10^5^ in 6-well plates. The following day, cells were infected with HCV at an MOI = 1 or left uninfected. For a positive control, the day prior to staining, cells were treated with lipopolysaccharide from *E. coli* 0127:B8 used at 5 µg/mL (LPS; *in vitro* LPS, Millipore-Sigma, L5024-10MG) for 3 h, followed by addition of Nigericin used at 167.5 µM (sodium salt, InvivoGen, tlrl-nig) and cultured overnight. At day four p.i., cell culture fluids were collected and cells trypsinised. Cells and culture fluids were centrifuged together at 400 x *g* for 5 min. Supernatant was discarded and cell pellets were resuspended in 200 µL of 30X caspase-1 probe in CM (same FAM-FLICA caspase-1 inhibitor kit as above) and incubated for 45 min at 37°C. Following incubation, an additional 400 µL of CM was added and cells were incubated for one additional hour. Cells were then centrifuged at 400 x *g* for 5 min and supernatants discarded. Cell pellets were then resuspended in 300 µL of apoptosis wash buffer and cells were fixed by adding 60 µL of fixative from the caspase-1 probe kit (ImmunoChemistry Technologies, product number 98). The cells were then incubated at room temperature in the dark for 20 min. Following incubation, cells were centrifuged as above and supernatant was discarded. Cells were resuspended in 500 µL PBS and stored at 4°C until analysis. Cells were run on a CytoFLEX flow cytometer (Beckman Coulter) and FAM-FLICA fluorescence was detected using the 525/40 detector on the 488 nm laser. Flow cytometry experiments performed on knockout cell lines were carried out concurrently and presented in different figures for analysis purposes. The percentage of cells that were positive for cleaved caspase-1 in both the infected and uninfected wild-type Huh-7.5 cells were used for comparison purposes in each of the figures that includes flow cytometry data from the knockout cells. Analysis of flow cytometry data was done using Kaluza software (version 2.1.1; Beckman Coulter). Gating strategy and analysis protocol are detailed below. 

### Virus Titre

Extracellular virus titre was determined by performing a 10-fold serial dilution of the virus stock, followed by infection in triplicate of Huh-7.5 cells in 8-well chamber slides that had been plated at a density of 5x10^4^ cells per well on the previous day. After infection, cells were incubated for two days and then fixed using acetone. Slides were stained for HCV core protein (Anogen, MO-I40015B) using the same dilutions as above and incubated at room temperature for 20 min. This was followed by incubation with goat anti-mouse Alexa Fluor^®^ 488, as described above. Slides were mounted with Vectashield containing DAPI. Virus titre was determined based on the number of foci present in the highest positive dilution and titre was then expressed as focus-forming units per millilitre (FFU/mL).

To measure intracellular infectious titre, both control and infected cells were harvested following a three-day infection at MOI = 0.1, pelleted by centrifugation for 5 min at 400 × *g* and re-suspended in 1 mL of CM. The re-suspended cells were then lysed by three cycles of freeze/thaw (3 min freeze, dry ice/methanol bath; 3 min thaw, 37°C water bath) and pelleted by centrifugation for 10 min at 1500 × *g*. Virus titres were determined as described above based on clarified supernatants. 

### Flow Cytometry Analysis

Controls employed to guide the analysis of flow cytometry data included unstained cells for each condition, LPS/Nigericin as a pyroptosis positive control, and heat shocked cells (65°C for 6 min, sufficient to kill the majority of cells) as a positive control for cell death. Uninfected, unstained wild-type Huh-7.5 cells acted as the negative control. Wild-type Huh-7.5 cells were always analyzed in parallel when performing this assay on knockout cells and comparisons were all made by comparing wild-type to knockout cells.

The overall cell population was gated on the forward vs. side-scatter plot using an inclusive gate as Huh-7.5 cells are heterogeneous and would be expected to vary in size even in the absence of HCV infection. Gates for subpopulations of cells were generated on forward vs. side-scatter plots using heat shock controls and uninfected cell controls and applied to LPS/Nigericin-treated controls. Two populations of cells were observed in our LPS/Nigericin treated control and these corresponded with the heat shocked cell population and the uninfected cell population. One population of cells was smaller (shifted down and to the left on the forward vs. side-scatter plot) and corresponded with the dead, heat-shocked cell sample. There was also a population of larger cells that had the same range of forward vs. side-scatter as the uninfected Huh-7.5 cells. As LPS/Nigericin stimulation is an established pyroptosis positive control, this two-population gating strategy guided further analysis of HCV-infected cell samples. Within the HCV-infected cell samples, there were the same two distinct cell populations observed in the LPS/Nigericin-treated control. The majority of the caspase-1 positive cells were located within the “small” population. However, it is important to note that not all the caspase-1 positive cells were in this population, and percent caspase-1 positivity and mean fluorescence intensity varied between conditions. In order to have sufficient numbers of caspase-1 positive cells that were representative of the whole sample, we recorded 10,000 events in the “small” population meaning at least 10,000 events were collected for each condition.

As dying cells take up the caspase-1 probe non-specifically, the heat shock population was used to set the caspase-1 fluorescence positivity threshold. Uninfected, unstained Huh-7.5 cells were used to gate out background fluorescence. These two gates were applied to the LPS/Nigericin-treated sample to ensure the gates were representative of caspase-1 positivity. The same gates were applied to all conditions for analysis. For clarification purposes, images of the gating strategy are also included in the supplementary materials (**Figure S1**). 

### Statistical Analyses

Statistical analyses for flow cytometry data were performed using the Analysis ToolPak in Microsoft Excel (2016). One-way ANOVA was used to compare conditions. Where p-values of < 0.05 were considered statistically significant. All flow cytometry statistical analysis was evaluated using at least three independent experiments.

Statistical analyses for viral titre data were performed using SPSS statistics 27. One-way ANOVA with Bonferroni multiple comparison test was used to compare between multiple groups. p-values < 0.05 were considered statistically significant.

## Results

### HCV Infection Induces Both Pyroptosis and Apoptosis, With Pyroptosis Preceding Apoptosis

Our previous research suggested that both apoptosis and pyroptosis were induced during HCV infection *in vitro* (24). To confirm this result and to elucidate the relative timing by which apoptosis and pyroptosis occur during HCV infection, infected (MOI=1) and uninfected cells, and culture fluids were compared for the presence of cleaved caspase-1, indicative of pyroptosis, and cleaved caspase-3, indicative of apoptosis, using Western blot analysis. This was accomplished by performing time-course experiments to compare cleaved caspase-1 and -3 expression over time. We first established that HCV core protein levels increased with subsequent days p.i. (**Figure 1A**) which was confirmed using fluorescent microscopy (**Figure 1C** **).** Cleaved caspase-1 peaked at day two p.i. and remained consistent at day three p.i. in culture fluids taken from HCV-infected cell cultures (**Figure 1A**). This coincided with an expected decrease of caspase-1 (full-length) levels in HCV-infected cells at days two and three p.i. (**Figure 1A**). Levels of cleaved caspase-1 were also shown to increase with increasing time p.i. in cells that remained adherent (**Figure 1C**). Apoptosis was initiated on day three p.i., indicated by a decrease in the level of caspase-3 in HCV-infected cells at days two and three p.i., while cleaved caspase-3 increased at day three p.i. (**Figure 1A**). Decreased expression of both caspase-1 and -3 coincided with an increase in their active, cleaved forms. These results were confirmed by fluorescence microscopy, using an antibody specific for cleaved caspase-3 and a probe specific for cleaved caspase-1, demonstrating increased caspase-1 and -3 activation in HCV-infected cells at three days p.i. when compared to uninfected controls (**Figure 1B**). Using flow cytometry to quantify the percentage of caspase-1-positive cells, we confirmed a significant difference in the percentage of caspase-1-positive cells between uninfected Huh-7.5 cells (~18%) and both HCV-infected (~50%) and LPS/Nigericin-treated (~45%) Huh-7.5 cells (**Figures 1D**, **S2**). To validate the use of caspase-1 and -3 as markers of pyroptosis and apoptosis, respectively, we also verified caspase-1 cleavage of downstream GSDM-D and caspase-3 cleavage of PARP. Cleavage of GSDM-D was detected using an antibody specific for cleaved GSDM-D, which was observed at a greater level in infected cells than uninfected controls (**Figure 1E**). We attempted to show this by Western blot analysis but we were unsuccessful with our current antibody. In accordance with our results showing the activation of caspase-3, cleaved PARP was undetectable by Western blotting, even with extended exposure, until day 3 p.i. (**Figure 1F**). This was also confirmed using fluorescence microscopy with an antibody specific for cleaved PARP which showed increased PARP cleavage in HCV-infected cells when compared to uninfected controls (**Figure 1G**). Overall, these results confirm involvement of both apoptosis and pyroptosis during HCV infection and suggest that pyroptosis precedes apoptosis during infection progression.

### NLRP3 Is One of the Sensors Involved in HCV-Induced Pyroptosis

**Figure 2:**
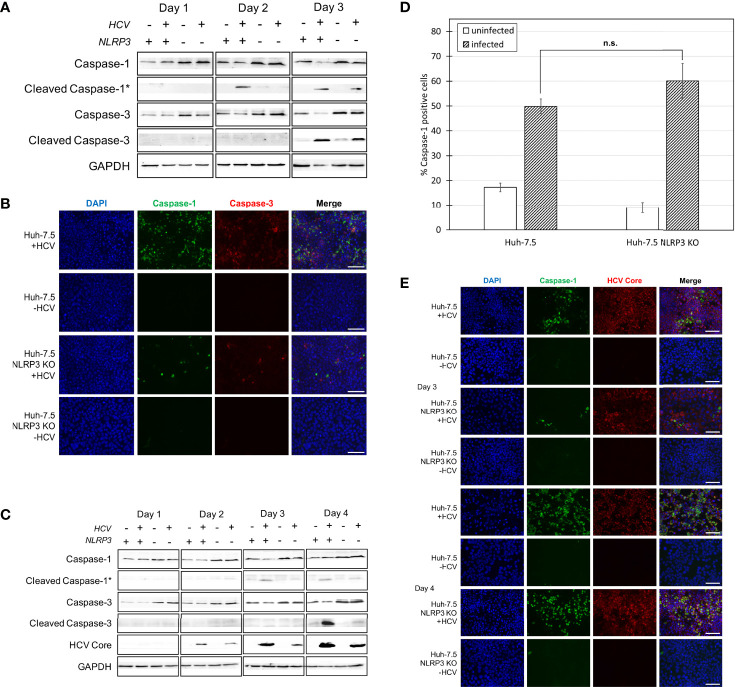
Caspase-1 and -3 activation in Huh-7.5 cells and Huh-7.5 NLRP3 KO cells. **(A, B)** Huh-7.5 cells or Huh-7.5 NLRP3 KO cells were infected with HCV at MOI = 1 or left uninfected. **(A)** At 1, 2, and 3 days p.i., cells and culture fluids were harvested for Western Blot analysis. Membranes were probed for pro-caspase-1, cleaved-caspase-1, pro-caspase-3, cleaved-caspase-3, and GAPDH. * indicates samples from cell culture fluids rather than cell lysates. **(B)** At 3 days p.i., cells were stained for cleaved caspase-1 (green) using a specific probe then fixed using acetone. Cells were subsequently stained using an antibody specific for cleaved caspase-3 (red). Nuclei were stained with DAPI (blue) for analysis by fluorescence microscopy. Scale bar, 100 μm **(C)** Huh-7.5 cells or Huh-7.5 NLRP3 KO cells were infected with HCV at MOI = 0.1 or left uninfected. At 1, 2, 3, and 4 days p.i., cells and culture fluids were harvested for Western Blot analysis. Membranes were probed for pro-caspase-1, cleaved-caspase-1, pro-caspase-3, cleaved-caspase-3, HCV core protein, and GAPDH. * indicates samples from cell culture fluids rather than cell lysates. **(D)** At day 4 p.i., cells were stained for cleaved caspase-1 using a specific probe and fixed using fixative from the caspase-1 probe kit. Cells were run on a CytoFLEX flow cytometer and data were analyzed using Kaluza analysis software. Data from NLRP3 KO cells are compared here to data from wild-type cells found in **Figure 1D**. Data are presented as the percent of total cells positive for caspase-1. p = n.s. **(E)** At 3 and 4 days p.i., cells were stained for cleaved caspase-1 (green) using a specific probe, then fixed using acetone. Cells were subsequently stained using an antibody specific for HCV core protein (red). Nuclei were stained with DAPI (blue) for analysis by fluorescence microscopy. Scale bar, 100 μm **(A–E)** Data are representative of three independent experiments.

Our previous work suggested that HCV-induced pyroptosis is mediated by the NLRP3 inflammasome since cell death was reduced when cells were treated with NLRP3 inhibitor, MCC950 (24). To further corroborate the involvement of NLRP3 in HCV-mediated pyroptosis, the CRISPR-Cas9 system was used to knock out NLRP3 in Huh-7.5 cells (**Figure S3**). Both wild-type Huh-7.5 and NLRP3 knockout (KO) cells were infected with HCV and Western blot analysis was performed on cells and cell culture fluids at one, two, and three days p.i. We observed decreased levels of caspase-1 on days two and three p.i. in infected NLRP3 KO cells compared to wild-type control cells. However, knockout of NLRP3 did not eliminate caspase-1 activation entirely (**Figure 2A**). Detection of cleaved caspase-1 in cell culture fluids was delayed until day three p.i. in culture fluids from NLRP3 KO cells compared to wild-type controls. Similar results were confirmed by fluorescence microscopy, with the infected NLRP3 KO cells displaying diminished caspase-1 activation compared to wild-type Huh-7.5 cells at day three p.i. (**Figures 2B**, **S4A**). Diminished cleaved caspase-1 expression was even more prominent when the Western blotting was performed on samples from an infection performed at an MOI = 0.1, likely due to the fact that activation of caspases is dependent on the extent of virus replication (**Figure 2C**). Western blot analysis and fluorescence microscopy revealed similar levels of cleaved caspase-3 at day three p.i. in both Huh-7.5 and NLRP3 KO cell lines when an MOI = 1 was used (**Figures 2A, B** **)** but, when an MOI = 0.1 was used, NLRP3 KO cells showed a reduction in levels of cleaved caspase-3 compared with wild-type cells at four days p.i. (**Figure 2C**). The difference in caspase-1 activation between Huh-7.5 cells and NLRP3 KO cells was clear at day three p.i. using Western Blot analysis and fluorescence microscopy. However, using flow cytometry, we showed no significant difference in the percentage of caspase-1 positive cells between HCV-infected Huh-7.5 and NLRP3 KO cells at day four p.i. (**Figures 2D**, **S4B**). When fluorescence microscopy was performed on cells four days p.i., there was also no detectable difference in caspase-1 activation between Huh-7.5 cells and NLRP3 KO cells (**Figure 2E**). Taken together, these results confirm involvement of NLRP3 in HCV-induced pyroptosis. The fact that caspase-1 activation was not completely eliminated at day three p.i. and was detected at similar levels to that of wild-type Huh-7.5 cells on day four p.i., indicates that other sensors upstream of caspase-1 may also be involved in HCV-induced pyroptosis or the NLRP3 KO was incomplete.

### Apoptosis Increases in the Absence of Pyroptosis

**Figure 3:**
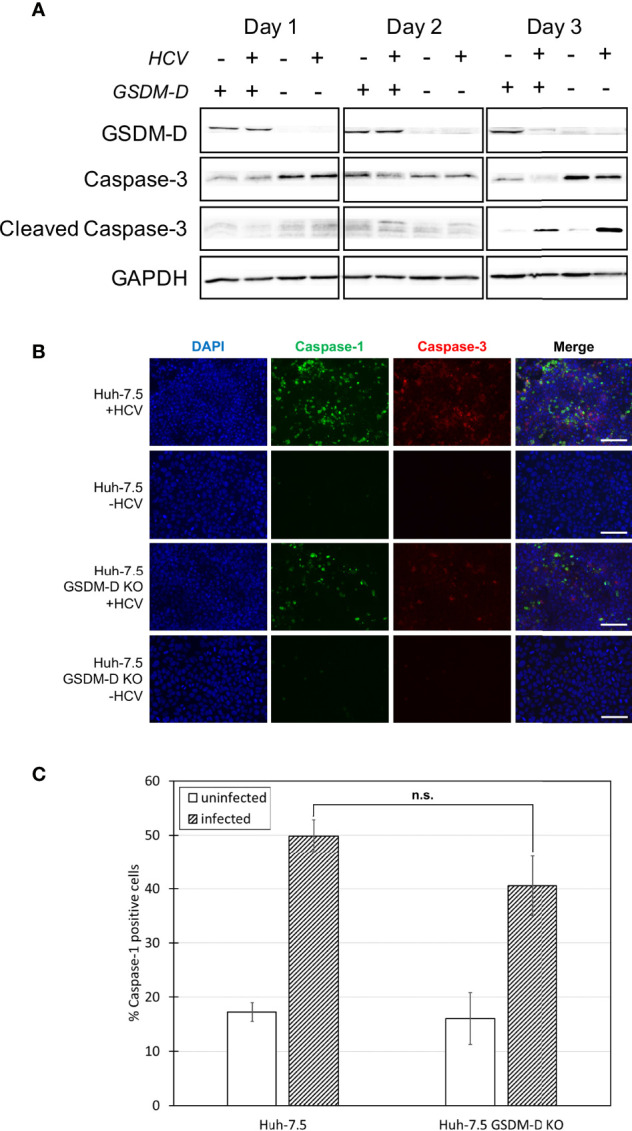
Analysis of caspase-1 and -3 levels in the absence of GSDM-D. Huh-7.5 cells or Huh-7.5 GSDM-D KO cells were infected with HCV at MOI = 1 or left uninfected. **(A)** At 1, 2, and 3 days p.i., cells and culture fluids were harvested for Western Blot analysis. Membranes were probed for GSDM-D, pro-caspase-3, cleaved-caspase-3, and GAPDH. **(B)** At 3 days p.i., cells were stained using a specific probe for cleaved caspase-1 (green), then fixed using acetone. Cells were subsequently stained using an antibody specific for cleaved caspase-3 (red). Nuclei were stained with DAPI (blue) for observation by fluorescence microscopy. Scale bar, 100 μm **(C)** At day 4 p.i., cells were stained for cleaved caspase-1 using a specific probe and fixed using fixative from the caspase-1 probe kit. Cells were run on a CytoFLEX flow cytometer and data were analyzed using Kaluza analysis software. Data from GSDM-D KO cells are compared here to data from wild-type cells found in **Figure 1D**. Data are presented as the percentage of total cells positive for caspase-1. p = n.s. **(A–C)** Data are representative of three independent experiments.

GSDM-D is a common component of the pyroptosis pathway across all sensors and is responsible for the final pore formation step leading to cell lysis (32–35). Early in our investigation, we raised the question as to what the outcome would be if one form of cell death was inhibited. Would the inhibition of pyroptosis cause cells to switch towards a different form of cell death, potentially indicating crosstalk between the two pathways (36–40)? To investigate the role of GSDM-D in HCV-induced pyroptosis and to investigate the likelihood of crosstalk between the pyroptotic and apoptotic pathways, CRISPR-Cas9 was utilised to generate Huh-7.5 cells lacking GSDM-D (**Figure 3A**). Wild-type and GSDM-D KO cells were infected with HCV at an MOI = 1 (**Figure 3A**). Cells and culture fluids were harvested from infected and uninfected conditions at one, two, and three days p.i.. Western blot analysis of GSDM-D KO cell lysates revealed that levels of both caspase-3 and cleaved caspase-3, indicative of apoptosis, increased at three days p.i. when compared to wild-type cells (**Figure 3A**). While the same increase of cleaved caspase-3 was not found when fluorescence microscopy was performed, there was a change in the caspase-3 staining morphology with more diffuse staining patterns (**Figures 3B**, **S5A**). These results suggest a change of cell death patterns induced by infection with HCV when GSDM-D has been knocked out whereby inhibition of one cell death pathway stimulated another. Results using fluorescence microscopy at three days p.i. and flow cytometry at four days p.i. both revealed non-significant differences in cleaved caspase-1 activation and percentage of cleaved caspase-1-positive cells, respectively (**Figures 3B, C**, **S5B**). We did notice a slight, but non-significant, decrease in the levels of cleaved caspase-1 in the GSDM-D KO cells when compared to wild-type Huh-7.5 cells. Lack of change in levels of cleaved caspase-1 is to be expected as caspase-1 is found upstream of GSDM-D in the pyroptosis pathway. Taken together, these results indicate that, in the context of HCV infection, there is crosstalk between pyroptotic and apoptotic pathways, with a shift from the pyroptotic to the apoptotic pathway in the event of pyroptosis inhibition.

### Pyroptosis Is Reduced in the Absence of Caspase-3-Mediated Apoptosis

**Figure 4:**
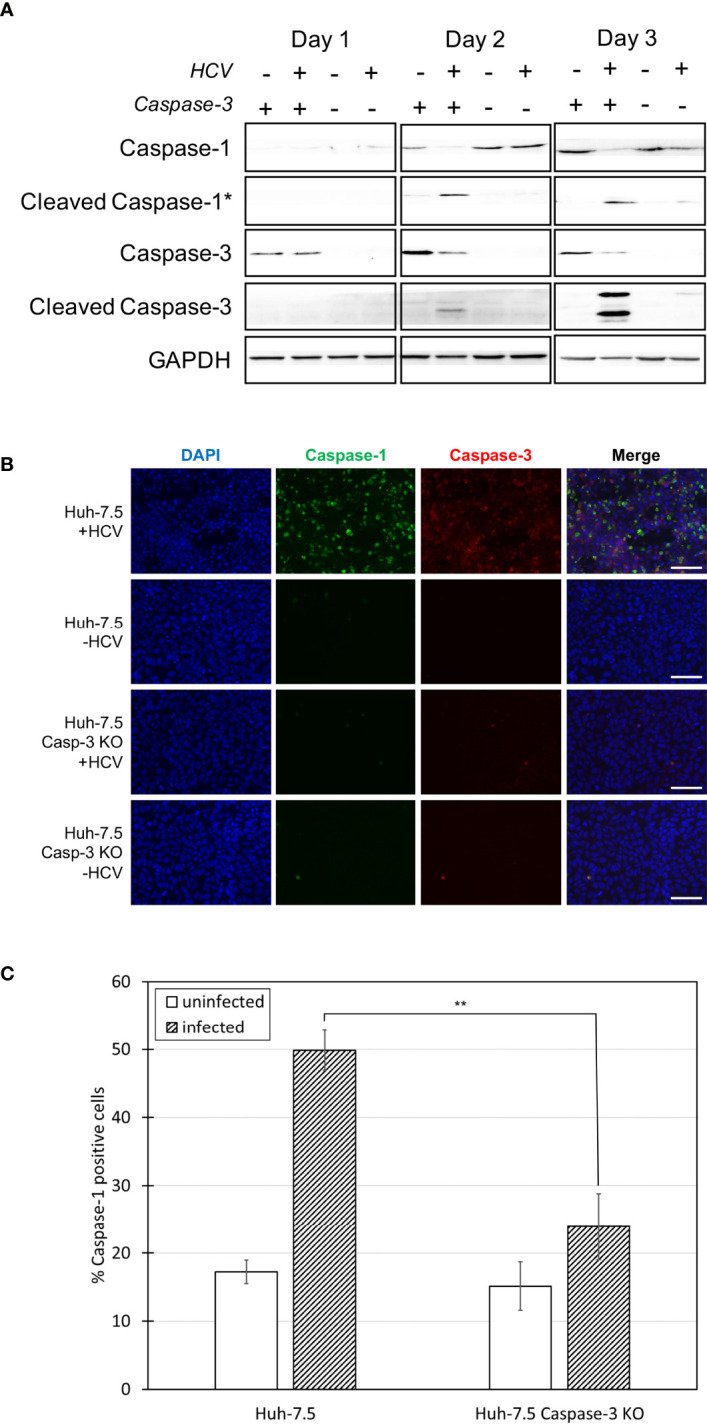
Comparison of caspase levels when apoptosis is inhibited. Huh-7.5 cells or Huh-7.5 caspase-3 KO cells were infected with HCV at MOI = 1 or left uninfected. **(A)** At 1, 2, and 3 days p.i., cells and culture fluids were harvested for Western Blot analysis. Membranes were probed for pro-caspase-1, cleaved-caspase-1, pro-caspase-3, cleaved-caspase-3, and GAPDH. * indicates samples from cell culture fluids rather than cell lysates. **(B)** At 3 days p.i., cells were stained using a probe specific for cleaved caspase-1 (green), then fixed using acetone. Cells were subsequently stained using a specific antibody for cleaved caspase-3 (red). Nuclei were stained with DAPI (blue) and analysis was by fluorescence microscopy. Scale bar, 100 μm **(C)** At day 4 p.i., cells were stained for cleaved caspase-1 using a specific probe and fixed using fixative from the caspase-1 probe kit. Cells were run on a CytoFLEX flow cytometer and data were analyzed using Kaluza analysis software and presented as the percent of total cells positive for caspase-1. Data from caspase-3 KO cells are compared here to data from wild-type cells found in **Figure 1D**. **p < 0.005. **(A–C)** Data are representative of three independent experiments.

More research regarding the phenomenon of crosstalk between cell death pathways has been reported in the literature in recent years (36, 37, 39–43). We investigated potential crosstalk between the apoptotic and pyroptotic pathways and examined whether pyroptosis increased if apoptosis was inhibited in the context of HCV infection. To do this, caspase-3 KO cells were generated using a CRISPR-Cas9 approach (**Figure 4A**). These cells were infected with HCV or left uninfected and levels of caspase-1 and -3 were compared to wild-type Huh-7.5 cells using Western blot analysis. Surprisingly, the level of cleaved caspase-1 in cell culture fluids was decreased in the HCV-infected KO cells compared to wild-type cells at both two and three days p.i. (**Figure 4A**). This reduction in the level of cleaved caspase-1 in the infected caspase-3 KO cells was also shown by fluorescence microscopy and indicates a reduction in the activation of the pyroptotic pathway (**Figures 4B**, **S6A**). Pro-caspase-1 levels in the caspase-3 KO cells remained at a level consistent with that of uninfected wild-type cells (**Figure 4A**). Reduction of the percentage of caspase-1 positive cells in the HCV-infected caspase-3 KO cell population (~25%) compared to wild-type HCV-infected Huh-7.5 cells (~50%) was confirmed and shown to be significant using flow cytometry at day four p.i. (**Figures 4C**, **S6B**). Reduced activation of pyroptosis-associated cleaved caspase-1 in the absence of caspase-3 may indicate caspase-3 is upstream of caspase-1 and further supports the existence of crosstalk between the apoptotic and pyroptotic pathways.

### Apoptosis and Pyroptosis Are Not Mutually Exclusive Within a Single Cell

**Figure 5:**
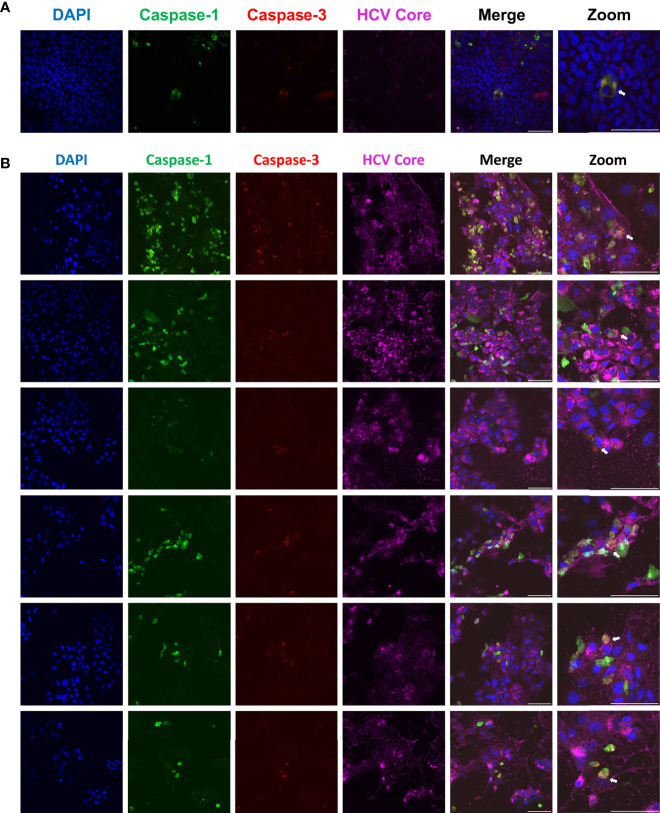
Single cells undergoing apoptosis and pyroptosis simultaneously. Huh-7.5 cells were infected with HCV at MOI = 1. At 3 days p.i., cells were stained for cleaved caspase-1 (green) using a probe and then fixed using acetone. Cells were subsequently stained using antibodies specific for cleaved caspase-3 (red) and HCV core protein (magenta). Nuclei were stained with DAPI (blue) for analysis by confocal microscopy. **(A)** The first observed instance of a double positive cell. **(B)** Subsequent experiments showing additional double positive cells. **(A, B)** Scale bar, 100 μm.

Throughout our study, a question emerged: could the pyroptotic and apoptotic pathways be activated simultaneously or are they mutually exclusive? By performing confocal microscopy on HCV-infected Huh-7.5 cells, we were able to capture images of rare single cells that were positive for active forms of both caspase-1 and caspase-3 (**Figure 5**; orange-yellow colour in the merged images). This indicates that multiple cell death pathways can be activated concurrently within the same cell. The first time this was observed is documented in **Figure 5A**. To confirm the finding of cells positive for both caspase-1 and -3, we subsequently performed further microscopy experiments to obtain additional examples of this observation (**Figure 5B**). At this point, we cannot say whether this observation represents concurrent cell death pathways, or if we are capturing cells in transition from one cell death pathway to another.

### Apoptosis and Pyroptosis Liberate Infectious Virus From Cells

**Figure 6:**
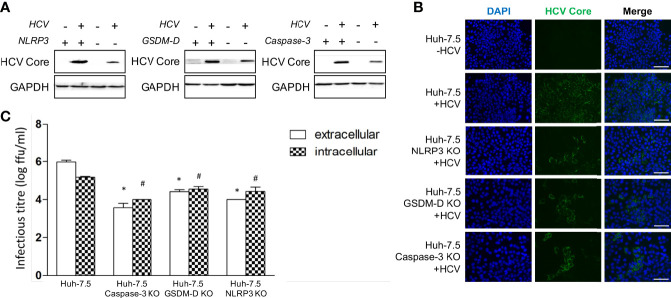
Apoptosis and pyroptosis involvement in HCV spread. Huh-7.5 cells or Huh-7.5 KO cells (NLRP3 or GSDM-D or caspase-3) were infected with HCV at MOI = 1 or left uninfected and allowed to propagate for 3 days following infection. **(A)** Cells were harvested for Western Blot analysis and membranes were probed for HCV core protein and GAPDH. **(B)** Cells were fixed using acetone and subsequently stained using an antibody specific for HCV core protein (green) and nuclei were stained with DAPI (blue) for analysis by fluorescence microscopy. Scale bar, 100 μm **(C)** Cells and cell culture fluids were harvested for determination of intra- and extracellular titres, respectively. Titre was determined using limiting dilution assay. * indicates p < 0.05 for differences in extracellular titre, # indicates p < 0.05 for differences in intracellular titre. **(A–C)** Data are representative of three independent experiments.

Early results in this study were accompanied by a finding of decreased HCV core protein levels in the NLRP3, GSDM-D, and caspase-3 KO cells when analysis was performed *via* Western Blot at day three p.i. (**Figure 6A**). A similar phenomenon was observed in the various KO cells when fluorescence microscopy was performed using an antibody against HCV core protein at day three p.i. (**Figure 6B**). To determine whether the lower levels of intracellular core protein correlated with decreased infectious virus production, we performed intra- and extracellular infectious titre assays on wild-type and KO cells. In our virus culture system, infectious virus propagates within Huh-7.5 cells and is subsequently released into culture fluids. When using wild-type Huh-7.5 cells, we typically see approximately 10-fold more infectious virus in the extracellular compartment by day three p.i. compared to the intracellular compartment. When we compared intra- and extracellular titres from the KO cell lines to the wild-type Huh-7.5 cells, we observed a significant reduction in both intra- and extracellular titres from the KO cell lines (**Figure 6C**). An important finding to note is that the KO cells displayed a change in the ratio of intra- to extracellular titre, with the intracellular titre being higher, suggesting an impairment of virus release. These results indicate that both apoptosis and pyroptosis are involved in, and necessary for, efficient virus release from infected cells *in vitro*. While our cell culture system cannot possibly recapitulate what happens with HCV infection *in vivo*, it is possible that programmed cell death plays a role that is beneficial to the virus and contributes to the pathogenesis associated with chronic HCV infection.

## Discussion

Despite availability of curative drugs to treat those infected with HCV, some individuals will still develop liver fibrosis/cirrhosis or hepatocellular carcinoma (16, 17). The mechanism behind the liver pathology associated with HCV infection, particularly following treatment with DAAs, remains a matter of significant interest. We previously proposed that pro-inflammatory programmed cell death, specifically pyroptosis, contributes to HCV-associated liver pathogenesis (24). Our current study provides further evidence and mechanistic findings to support the potential role for virus-induced pyroptosis to promote inflammation in the liver.

The current study demonstrated that, during HCV infection, pyroptosis is initiated earlier than apoptosis. We confirmed the involvement of the NLRP3 inflammasome in HCV-induced pyroptosis although involvement of other sensors cannot be ruled out. We also demonstrated crosstalk between the apoptosis and pyroptosis pathways within the context of HCV infection *in vitro*. This work also contributed to the understanding of HCV pathogenesis by demonstrating that HCV release was impaired in cell lines with various components of the pyroptotic or apoptotic pathways knocked out.

It was unsurprising that pyroptosis increased with increasing time p.i. as pyroptosis can be explained in a circular manner with the initiation of pyroptosis in one cell seeming to contribute to that of nearby cells (1, 24, 44, 45). Initiation of pyroptosis prior to apoptosis may indicate that pyroptosis is a contributing factor in the pathology associated with HCV-induced liver disease. This is particularly relevant as it is widely accepted that liver fibrosis/cirrhosis associated with chronic infection is also associated with substantial inflammation (46). Future research should confirm this phenomenon *in vivo* during chronic infection. As both apoptosis and pyroptosis occur over the course of infection, these two cell death pathways likely play important and complementary roles, contributing to the progression of liver disease during chronic HCV infection.

**Figure 7:**
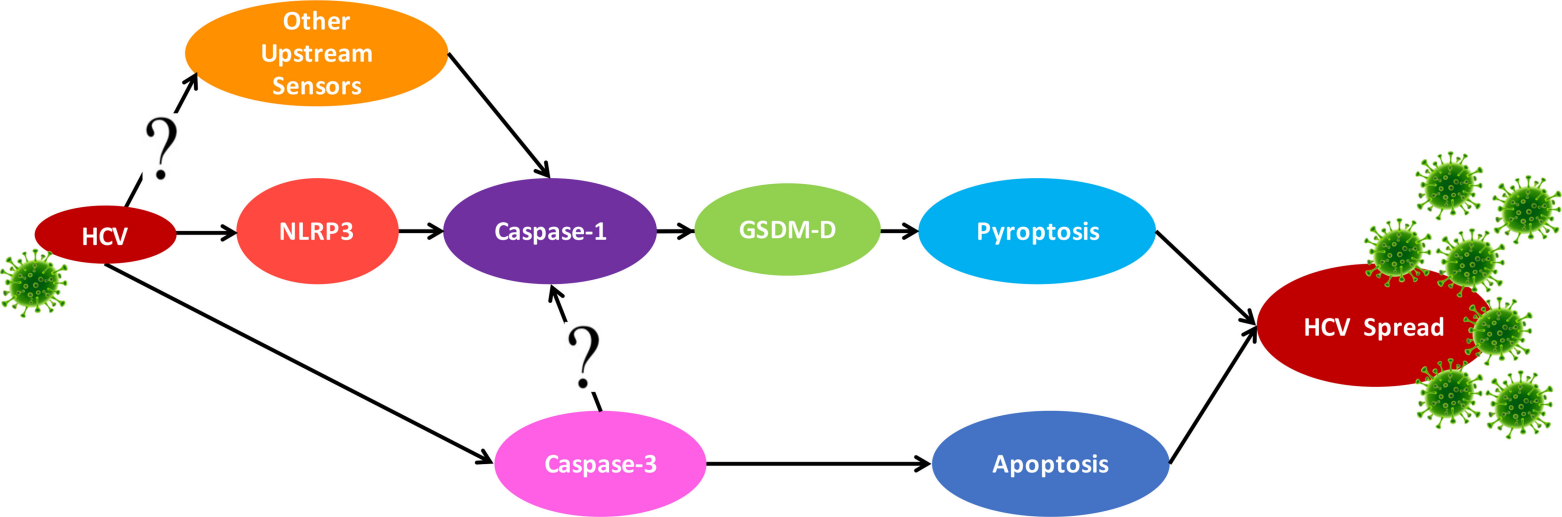
Proposed crosstalk between the apoptosis and pyroptosis pathways. Schematic for the proposed crosstalk between the apoptosis and pyroptosis pathways and how they may contribute to HCV spread. HCV infection can trigger pyroptosis *via* the NLRP3 inflammasome pathway but likely also triggers inflammasome formation with other sensors. Apoptosis is also activated in the presence of HCV but this is reduced when pyroptosis is blocked.

Knockout of NLRP3 decreased caspase-1 activation, although it was not entirely eliminated during HCV infection. This confirms the role of NLRP3 in HCV-induced pyroptosis but suggests it may not be the only sensor involved (**Figure 7**). Several sensors, including, but not limited to, RIG-I (47), IFI16 (1), NLRP9b (48), and AIM2 (49), have been shown to form inflammasomes during viral infection (50). It has been previously established that RNA viruses typically induce the NLRP3 inflammasome (51) while most DNA viruses induce AIM2 or IFI16 inflammasomes [reviewed in reference (50)]. However, recent research does not always support this RNA virus-NLRP3/DNA virus-AIM2 dogma (52–54). Close examination reveals that some viruses can actually activate multiple sensors, so future studies should investigate other sensors that may be triggered during HCV infection. We clearly demonstrate that HCV induces pyroptosis *via* the NLRP3/caspase-1 pathway, but this activation of caspase-1 may also rely on other sensors.

The apoptotic and pyroptotic pathways were traditionally thought to be mutually exclusive. However, recent research has indicated there may be significantly more crosstalk than originally perceived (36, 37, 39–43). Our findings demonstrate noteworthy crosstalk between the apoptotic and pyroptotic pathways during HCV infection (**Figure 7**). In the absence of GSDM-D, caspase-3 activation was increased, indicating a switch from pyroptotic cell death to apoptotic. This is in agreement with a recent study by Tsuchiya et al. (55) that demonstrates caspase-1 initiates apoptosis in the absence of GSDM-D, which is accompanied by an increase in the expression of caspase-3 and subsequent apoptotic cell death. This is also supported by an earlier study that used GSDM-D KO macrophages to show these cells do not die by pyroptosis, but rather by a mechanism morphologically similar to apoptosis (56). An additional study by Schneider et al. (57) showed that in the absence of GSDM-D, an alternate form of cell death was induced that utilised apoptosis-associated caspases (3 and 8) but was morphologically distinct from traditional apoptosis. As indicated in our results, we observed a change in the morphology of the cleaved caspase-3 staining by fluorescence microscopy, rather than a clear increase as seen by Western blot. This may indicate a phenomenon more similar to the examples above, whereby, in the absence of GSDM-D, cells underwent a form of caspase-3-dependent cell death morphologically distinct from apoptosis observed in wild-type cells.

In our study, caspase-3 KO cells showed decreased caspase-1 activation, suggesting that caspase-3 may somehow play an important role in pyroptosis induction. For example, apoptotic bodies that are not cleared by macrophages can undergo secondary pyroptosis *via* caspase-3-mediated cleavage of GSDM-E, resulting in cell death that is morphologically similar to pyroptosis (58). However, this does not account for the decrease in caspase-1 activation when caspase-3 was knocked out (**Figure 4**). It is known that GSDM-D can be cleaved by caspase-3 (59, 60) and it is conceivable, therefore, that in the absence of caspase-3, there would be less GSDM-D being cleaved into its active form. Here we propose that multiple sensors can trigger the pyroptosis pathway in response to HCV, that caspase-3 can promote caspase-1 activation, and that apoptosis is initiated in the absence of GSDM-D (**Figure 3**). Investigation into the mechanisms of crosstalk between the apoptotic and pyroptotic pathways and which proteins are involved is therefore an important area of future research.

To our knowledge, simultaneous activation of both the apoptotic and pyroptotic pathways in one cell during HCV infection has never before been documented (**Figure 5**). Significant time has been invested in optimising flow cytometry protocols to quantify what proportion of the cell population display both caspase-1 and -3 activation, although we have yet to establish an ideal protocol.

The NLRP3, GSDM-D, and caspase-3 KO cell lines showed a decrease of both extracellular and intracellular titre upon infection compared to wild-type cells, indicating that programmed cell death is necessary for efficient HCV propagation (**Figure 6**). The inversion of the ratio between extracellular and intracellular titre in the KO cells compared to wild-type cells is important to note. This finding suggests that knocking out important cell death pathway components can affect the quantity of infectious virions able to exit the cell, with a larger proportion remaining “stuck” within the cells. Although HCV has classically been regarded as a non-lytic virus, these results suggest cell lysis may, in fact, be part of the HCV lifecycle. Taken together, these results suggest programmed cell death is an important part of efficient HCV propagation and may represent a mechanism by which HCV induces liver disease *in vivo*.

It remains generally unclear whether pyroptosis in the context of viral infection is of benefit to the host, acting as an innate anti-viral response, or used as a mechanism of pathogenesis, exploited by the virus to induce disease. Classically, apoptosis has been considered an innate antiviral pathway activated by the host immune system to limit virus replication and propagation (61). However, under certain circumstances, and as identified in this study, apoptosis and pyroptosis have been found to promote both an increase of viral replication (3, 62) and the release of virus from infected cells, thereby potentially promoting pathogenesis (63, 64). It is likely that both ideas are correct in the context of different viruses. Some viruses exploit cellular pathways to induce efficient propagation and pathogenesis while others inhibit these pathways so efficient replication can occur without stimulating the innate immune responses of the cell. Here we argue that HCV utilizes the apoptotic and pyroptotic pathways of the host for efficient propagation. This is aligned with the observation that the M2 protein of influenza A virus induces pyroptosis and that efficient propagation is hindered in the absence of the M2 protein (13). Similarly, caspase-3 activation by Bovine Herpesvirus 1 results in programmed cell death accompanied by efficient virus release (65).

As mentioned, reports demonstrating crosstalk between cell death pathways have been accumulating in recent years. Some of this research has focused on PANoptosis (36, 38, 66), the activation of pyroptosis, apoptosis, and necroptosis simultaneously within a given condition, accompanied by formation of a PANoptosome [protein complex containing RIPK1, RIPK3, NLRP3, ASC, Z-DNA-binding protein 1 and caspases 1, 6, and 8; reviewed in reference (67)]. PANoptosis has been observed in other systems with various pathogens including influenza and several species of fungi (37, 38). At this time, we cannot confirm that PANoptosis is occurring within our HCV infection system without the examination of necroptosis components; however, this would be a logical and interesting follow-up of the current work.

Our study utilised a cell culture-adapted strain of HCV, JFH1_T_, and human hepatoma-derived Huh-7.5 cells. While this system has been invaluable for identifying drug targets and understanding the HCV life cycle [reviewed in reference (68)], it, as with any cell culture system, does impose inherent limitations on the study. JFH1_T_ is unlike other HCV patient isolates in that it replicates in cell culture without adaptive mutations or modifications of the cells (69). Huh-7.5 cells are cancer-derived and are widely known to have deficiencies in pathways involved in innate immunity (70, 71). Due to these limitations, any findings presented here should be verified by using *in vivo* animal studies and biopsies from HCV-infected patients.

Despite the *in vitro* limitations discussed above, the current findings should be considered within a larger context, taking into account what is already known in relation to programmed cell death in animal studies and human samples. This study cannot definitively say that these forms of programmed cell death are occurring in the livers of infected individuals. However, there is some literature that suggests this is the case (22, 72–75). In addition, there is well-documented liver inflammation in the context of HCV infection, even after treatment with DAAs (76–78) and it is likely that pyroptotic cell death is one of the driving factors behind this inflammation. Higher levels of circulating pyroptosis-associated inflammatory cytokines (IL-18 and IL-1*β*) have been documented in patients infected with HCV (18, 79) but it is important to note that pyroptosis itself (i.e. inflammatory cell death) has never been documented in human liver samples directly. Moving forward, it will be crucial to identify whether pyroptosis occurs in the livers of HCV-infected patients to identify whether the pathology we identified is physiologically/biologically relevant.

In conclusion, we demonstrated that pyroptosis is initiated prior to apoptosis during HCV infection, with NLRP3 and possibly other sensors involved in HCV-induced pyroptosis. There appears to be substantial crosstalk between the apoptotic and pyroptotic pathways, at least in the context of HCV infection (**Figure 7**). The role of this crosstalk and the mechanism by which it occurs remain to be elucidated. We show here that programmed cell death plays a role in efficient HCV propagation and, therefore, pathogenesis of HCV. Since curative drugs are available to treat HCV, future studies should address the role of programmed cell death *in vivo* as well as identify drug candidates whose mechanism of action is against one of these pathways in order to prevent the pathogenic effects of HCV that are not always mitigated with elimination of the virus (18, 76–78).

## Data Availability Statement

The raw data supporting the conclusions of this article will be made available by the authors upon request.

## Author Contributions

Conceptualization, HW, LW, KH, MG, and RR. Methodology, HW, LW, CG, CC. Investigation, HW performed fluorescence microscopy. HW and CG performed flow cytometry experiments. LW created knockout cell lines and performed Western blot analysis and titre experiments. Formal analysis, HW and CG performed the statistical analysis for flow cytometry experiments. LW performed statistical analysis for titre experiments. Writing—original draft, HW and LW. Writing—review and editing. HW, KH, MG, and RR. Funding acquisition, RR. Resources, KH and RR. Supervision, KH and RR. All authors read and revised the manuscript and gave final approval for publication.

## Funding

HW received the Memorial University School of Graduate Studies Aldrich Award, a Memorial University Faculty of Medicine Dean’s Fellowship (MSc and PhD), a Canadian Institutes of Health Research Banting and Best Canada Graduate Scholarship—Masters, and a Canadian Network on Hepatitis C Virus Doctoral Fellowship. This research was supported by research grants to RR (FRN#PJT-159675) and KH (FRN#PJT-153238) from the Canadian Institutes of Health Research, as well as to RR from the Medical Research Foundation, Faculty of Medicine, Memorial University. The funders had no role in study design, data collection and analysis, decision to publish, or preparation of the manuscript.

## Conflict of Interest

The authors declare that the research was conducted in the absence of any commercial or financial relationships that could be construed as a potential conflict of interest.

## Publisher’s Note

All claims expressed in this article are solely those of the authors and do not necessarily represent those of their affiliated organizations, or those of the publisher, the editors and the reviewers. Any product that may be evaluated in this article, or claim that may be made by its manufacturer, is not guaranteed or endorsed by the publisher.
