# Supplementary material for: Crosstalk Between Pyroptosis and Apoptosis in Hepatitis C Virus-induced Cell Death
Source: Front Immunol. 2022 Feb 14;13:788138. doi: 10.3389/fimmu.2022.788138 (PMC8882739; doi:10.3389/fimmu.2022.788138)
Supplement: Supplementary file 1 [file DataSheet_1.pdf]

Supplementary Material

Crosstalk Between Pyroptosis and Apoptosis in Hepatitis C Virus-induced Cell Death

Hannah L. Wallace[1†], Lingyan Wang[1†], Cassandra L. Gardner[1], Christopher P. Corkum[2], Michael D. Grant[1], Kensuke Hirasawa[1] and Rodney S. Russell[1*]

[1] Immunology and Infectious Diseases, Division of Biomedical Sciences, Faculty of Medicine, Memorial University, St. John's, NL, Canada

[2] Confocal Imaging/Flow Cytometry Unit, Medical Laboratories, Faculty of Medicine, Memorial University, St. John's, NL, Canada

[†] These authors have contributed equally to this work and share first authorship.

*** Correspondence:**
Rodney S. Russell, rodney.russell@med.mun.ca

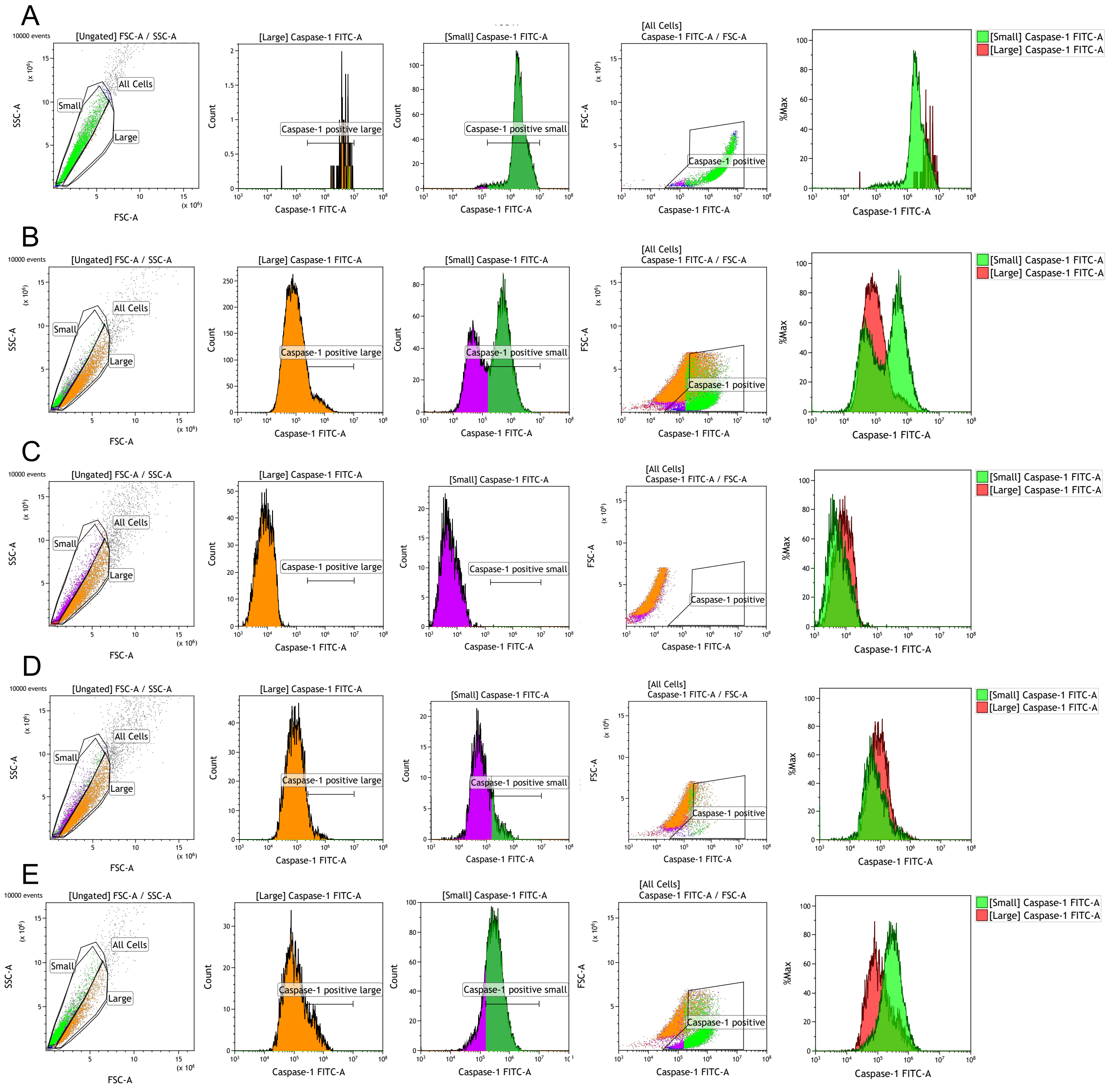

Figure S1 | Flow Cytometry gating strategy. All gating strategies were based on wild-type Huh-7.5 cells, for which representative examples are shown here. Forward versus side scatter plots were used to gate cell populations. Histograms were used to gate positivity for each cell population of interest. Forward scatter versus caspase-1 probe fluorescence intensity was also used to confirm gated positivity. A histogram for percent max was used to visualize mean fluorescence intensity (MFI) of the two cell populations of interest. **(A)** Heat shocked cell condition, stained **(B)** LPS/Nigericin treatment, stained **(C)** Uninfected, unstained **(D)** Uninfected, stained **(E)** Infected, stained. **(A-E)** For each condition, a minimum of 10,000 events were collected. For HCV-infected cell conditions (Huh-7.5 cells and KO cells), 10,000 events were collected in the "small" population, meaning that far more than 10,000 events were collected in total. See methods section for detailed explanation of the gating strategy.

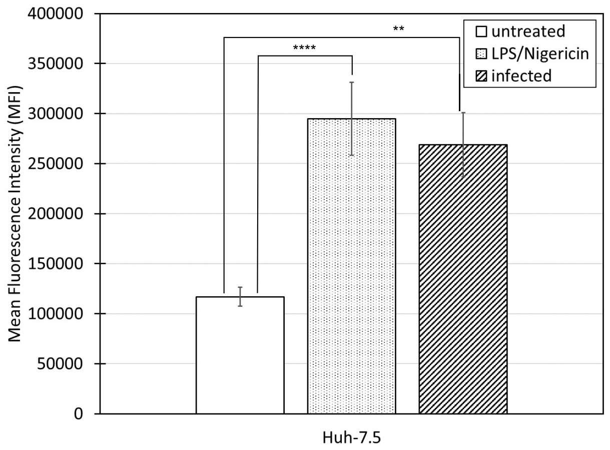

Figure S2 | Caspase-1 activation in Huh-7.5 cells. Huh-7.5 cells were infected with HCV at MOI = 1 or left uninfected. At day 4 p.i., cells were stained for cleaved caspase-1 using a specific probe and fixed using fixative from the caspase-1 probe kit. Cells were run on a CytoFLEX flow cytometer and data were analyzed using Kaluza analysis software. Results are presented as mean fluorescence intensity. ** $p < 0.005$; **** $p < 0.0001$.

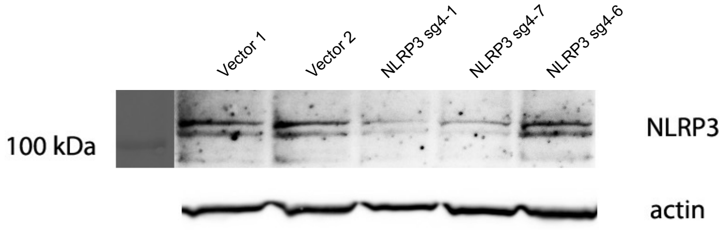

Figure S3 | Generation of NLRP3 KO cells. CRISPR-Cas9 was used to generate Huh-7.5 cells with NLRP3 knocked out. Two clones showed reduced levels of NLRP3. The NLRP3 sg4-7 clone was used for all experiments that included NLRP3 KO cells.

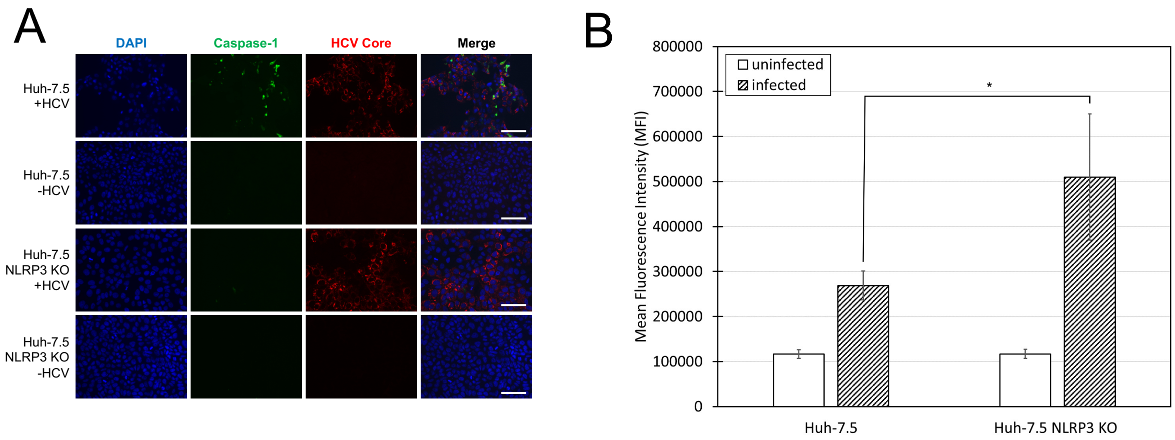

Figure S4 | Analysis of caspase-1 activation in NLRP3 KO cells. Huh-7.5 cells or Huh-7.5 NLRP3 KO cells were infected with HCV at MOI = 1 or left uninfected. **(A)** At 3 days p.i., cells were stained using a specific probe for cleaved caspase-1 (green), then fixed using acetone. Cells were subsequently stained for HCV core protein (red) using a specific antibody. Nuclei were stained with DAPI (blue) for observation by fluorescence microscopy. Scale bar, 100 μm. **(B)** At day 4 p.i., cells were stained for cleaved caspase-1 using a specific probe and fixed using fixative from the probe kit. Samples were run on a CytoFLEX flow cytometer and data was analyzed using Kaluza analysis software. Results are presented as mean fluorescence intensity. Data from NLRP3 KO cells are compared here to data from wild-type cells found in **Figure S1**. *, $p < 0.05$. **(A,B)** Data are representative of three independent experiments.

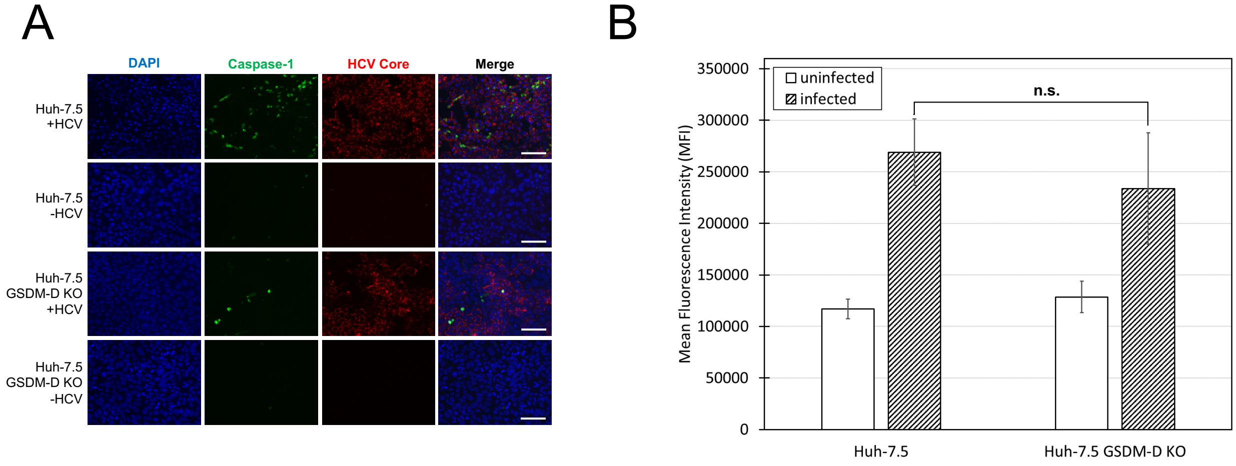

Figure S5 | Caspase-1 activation in HCV-infected GSDM-D KO cells. Huh-7.5 cells or Huh-7.5 GSDM-D KO cells were infected with HCV at MOI = 1 or left uninfected. **(A)** At 3 days p.i., cells were stained using a specific probe for cleaved caspase-1 (green), then fixed using acetone. Cells were subsequently stained for HCV core protein (red) using a specific antibody. Nuclei were stained with DAPI (blue) for observation by fluorescence microscopy. Scale bar, 100 μm. **(B)** At day 4 p.i., cells were stained for cleaved caspase-1 using a specific probe and fixed using fixative from the caspase-1 probe kit. Cells were run on a CytoFLEX flow cytometer and data was analyzed using Kaluza analysis software. Results are presented as mean fluorescence intensity. Data from GSDM-D KO cells are compared here to data from wild-type cells found in **Figure S1**. p = n.s. **(A,B)** Data are representative of three independent experiments.

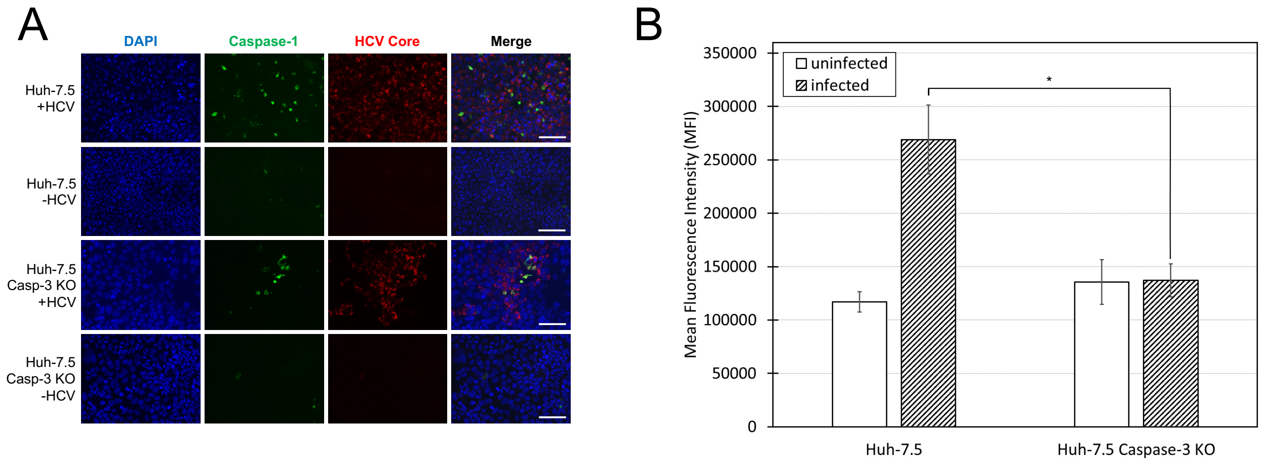

Figure S6 | Caspase-1 activation of wild-type Huh-7.5 cells and caspase-3 KO cells infected with HCV. Huh-7.5 cells or Huh-7.5 caspase-3 KO cells were infected with HCV at MOI = 1 or left uninfected. **(A)** At 3 days p.i., cells were stained using a specific probe for cleaved caspase-1 (green), then fixed using acetone. Cells were subsequently stained for HCV core protein (red) using a specific antibody. Nuclei were stained with DAPI (blue) for observation by fluorescence microscopy. Scale bar, 100 μm. **(B)** At day 4 p.i., cells were stained for cleaved caspase-1 using a specific probe and fixed using fixative from the caspase-1 probe kit. Cells were run on a CytoFLEX flow cytometer and data was analyzed using Kaluza analysis software. Data are presented as mean fluorescence intensity. Data from caspase-3 KO cells are compared here to data from wild-type cells found in **Figure S1**. *, $p < 0.05$. **(A,B)** Data are representative of three independent experiments.
